# Beyond the Local Agenda: International Perspectives in Community-based Arts and Health

**DOI:** 10.1080/17533015.2013.841336

**Published:** 2013-09-23

**Authors:** Sarah Atkinson, Mike White

**Affiliations:** ^a^ Centre for Medical Humanities, Durham University, Caedmon Building, Leazes Road, Durham, DH1 1SZ, UK

## 

The contemporary challenges to health and well-being expose a tendency towards two contrasting modes of engagement and understanding. On the one hand, policy analysts observe an individualisation of responsibility and blame for the production of health and ill-health; on the other, attention to social determinants, inequalities and globalisation locate the production of health and ill-health within complex relationships shaped across both space and time. Whilst arts and health activities function within both modes, their local and fragmented nature has meant that, to date, little research attention has been accorded within the second mode to the potential value to the field of an international focus.

The papers in this special issue on international perspectives in community-based arts and health offer a first set of explorations that both tease out the riches from comparative research and, hopefully, may provoke further engagements of research at this scale. The opening paper, by Parkinson and White, lays out the issues confronting the field of arts and health in tackling health inequalities both nationally and internationally. The authors reflect on the histories and current trajectories of arts and health networks across a number of English and non-English speaking nations, thereby articulating the issues in building the strength of the grassroots traditions of arts and health towards greater impact. Wright and colleagues pick up the theme of how arts and health projects may generate change through a comparative study across different sites in an Australian initiative, hART, for disconnected young people. They position the project's impact as emergent from productive tensions across seven domains of change, arguing that how arts practice animates these domains is crucial to impact. The emphasis from this single country study on the management of tension across specific domains offers a general model that demands validation in other country settings. Continuing with the nature of impact, Jensen examines the factors that influence the ability of arts and health organisations to enhance their participants' wellbeing through comparison of two projects in the United Kingdom and Denmark that aim to impact on social capital and identity as elements of well-being. Jensen emphasises the importance for arts and health organisations of a supportive policy environment with strategies for sustainability. The fourth paper shifts the focus from impact onto the nature of arts and health practice itself. Raw and Mantecón compare artists' practice across the North of England and Mexico City and, despite little shared history, language or policy priorities, find a convergence in practice captured through six elements. Exposing the commonalities in practice for community-based arts and health across very different settings simultaneously exposes the potential for international cooperation in professional development, institutional support and alliances for advocacy. Wilkinson and colleagues complement this attention to practice in demonstrating the potential for volunteer, peer involvement in arts and health projects, in this case in arts activities facilitated by seniors for other seniors who are socially isolated or in underserved areas in Canada. The final paper takes the contributions of arts and health into the heart of international development for health and well-being. Pavlicevic and Impey draw on music-based projects in the conflict and post-conflict settings of Lebanon and South Sudan to offer a framework based on what they call ‘deep listening’ that opens possibilities for a more culturally inclusive approach to health and well-being that can reintegrate self, place and community.

The first paper by Parkinson and White describes recent calls for a re-imagining of public health, the last for a re-imagining of international development for health and well-being. All six papers each offer different examples of how attention to international comparisons in arts and health research may contribute in responding to such calls. Whilst arts and health projects may appear to have only limited and local reach, placed into a framework of social determinants, inequalities and globalisation, international comparison and collaboration can facilitate a more truly radical potential for the field.

